# Integrated Rheumatology-Gastroenterology Clinic: An Innovative Organisation for Patients with Multiple Autoimmune Diseases

**DOI:** 10.5334/ijic.9292

**Published:** 2025-11-07

**Authors:** Sarah Holm Junge Jensen, Michal Frumer, Eileen Dorte Shanti Connelly, Rene Østgård, Henning Glerup, Kate Denby, Anja Leth Egsgaard, Charlotte Weiling Appel

**Affiliations:** 1 Medical Diagnostic Center, University Clinic for Innovative Patient Pathways, Regional Hospital Central Jutland, Silkeborg & Viborg, Denmark; 2 Department of Research, Horsens Regional Hospital, Denmark; 3 Research Clinic for Functional Disorders and Psychosomatics, Aarhus University Hospital, Aarhus, Denmark; 4 Department of Medicine, Goedstrup Hospital, Herning, Denmark

**Keywords:** integrated care, collaborative care, outpatient clinic, continuity of care, autoimmune diseases, multimorbidity

## Abstract

**Introduction:**

Patients with multiple autoimmune diseases lack continuity of care due to increasing specialisation and siloed practice in healthcare. Despite improvements in quality, this organisation has led to fragmented patient pathways, as related diseases are treated separately. Limited research has investigated approaches to integrate care for patients with co-occurrent Inflammatory Joint Disease and Inflammatory Bowel Disease, with minimal emphasis on the patient perspective. The aim was to describe the Rheumatology-Gastroenterology Clinic (ReGa), characterise its population, and investigate patient experiences.

**Description:**

A Danish outpatient clinic combining rheumatology and gastroenterology.

**Results:**

During the study period, 54 patients attended the ReGa clinic. Prior to integration, these patients had an average of 29.6 outpatient visits. With most working-age patients, this frequent attendance poses individual and societal challenges. Based on Haggerty et al.’s definition of continuity of care, relational elements emerged as particularly important for patients but not independent of informational and management factors.

**Conclusion:**

The integrated approach was experienced to improve continuity of care for patients with multiple autoimmune diseases. The findings highlight the potential to bridge healthcare gaps and address challenges arising from organisational structures shaped by specialisation and compartmentalisation of knowledge. This approach may also benefit other patient groups with comorbid conditions.

## Introduction

Over the decades, the Danish healthcare system has become specialised and centralised with the aim of offering patients treatment of the highest quality [1,2,3]. However, increasing specialisation and siloed knowledge practice has led to incoherent and fragmented patient pathways for some patients with multiple chronic diseases, as care is typically divided among several departments [2,4,5]. For patients managing multiple autoimmune diseases, specialisation poses particular challenges, requiring them to see different specialists in separate clinics despite the interrelatedness of their conditions [6,7,8,9]. As a result, this division hinders cooperation and coordination, which remain essential yet difficult to achieve [10,11,12].

Globally, at least one autoimmune disease affects 3–5% of the population [13]. The prevalence has increased significantly in recent decades and is expected to continue [13,14,15]. Inflammatory Joint Disease (IJD) and Inflammatory Bowel Disease (IBD) are among the most common autoimmune diseases. They often coexist, and a diagnosis of one increases the risk of developing the other [16]. Rheumatic diseases are among the most prevalent extraintestinal manifestations of IBD. Spondyloarthritis (SpA) is diagnosed in up to 13% of IBD patients [17]. Although IBD and SpA are clinically distinct, they share underlying inflammatory pathways. However, the exact pathogenetic mechanisms remain largely unknown [18]. What is clear is that the co-occurrence of these conditions significantly disrupts patients’ personal and occupational lives, as well as complicating healthcare treatment [13]. The treatment is complicated by factors such as the under-recognition of other autoimmune diseases, lack of standardised outcome measures, and inconsistent treatment goals across medical specialties, often leading to under-diagnosed and under-treated comorbidities. These difficulties call for a strategic reorganisation of care. Effective management of coexisting autoimmune diseases demands an integrated treatment approach, leveraging medical expertise across specialities.

In a Danish context, organisational restructuring has been recommended to address the complexities of medical specialisation by promoting collaboration between specialities [11,19]. Despite this, literature on integrated care for patients with multiple autoimmune diseases is sparse, particularly concerning combined treatment approaches across specialties. Current studies mainly examine the combination of dermatology and rheumatology involving the treatment of psoriasis and psoriatic arthritis [20,21,22,23,24,25,26,27,28,29,30]. Research on the integration of rheumatology and gastroenterology in autoimmune diseases is much less common [31,32,33,34]. All studies focus on early detection and clinical outcomes, and methodology and approach are rarely explained. In addition, few studies include patient perspectives through surveys [21,24,28], but no qualitative studies are available and patient involvement is lacking in this research area [35]. This research gap impedes a holistic understanding of patients’ experiences and needs and may lead to treatment and care strategies that are not fully adapted or responsive to the complexities of living with multiple autoimmune diseases.

Recognition of these unmet needs gave rise to the establishment of the Rheumatology-Gastroenterology clinic (ReGa) at Silkeborg Regional Hospital, Denmark, where patients with suspected or coexisting inflammatory joint and bowel disease were offered a multidisciplinary assessment with the purpose of ensuring appropriate diagnosis, non-interacting medical treatment, and improved continuity of care. This article aims to describe the ReGa clinic, characterise the attending population, and investigate patient experiences with the clinic’s innovative organisation.

## Description of the Care Practice

### Context

The ReGa clinic was established in 2018 at the Diagnostic Centre (now part of Medical Diagnostic Centre), Silkeborg Regional Hospital, Denmark. The Diagnostic Centre includes both the Gastroenterology Clinic, which receives patients from Silkeborg and the surrounding area, and the Rheumatology Clinic, which has a specialised diagnostic function in the Central Denmark Region (one of Denmark’s five regions). The Danish health care system is financed through taxes with universal access to general practice and hospitals. Most chronically ill patients regularly visit their general practitioner, and complex cases may also be managed at specialised outpatient clinics.

### Description of the Rheumatology-Gastroenterology Clinic ReGa

Information on the ReGa clinic was based on observations and interviews with two specialists who founded the clinic, one gastroenterologist and one rheumatologist. The description was inspired by the Template for Intervention Description and Replication (TIDieR) checklist and guide [36] with the aim of sufficient reporting. A detailed description is reported in Table 1.

**Table 1:** Description of The Rheumatology-Gastroenterology clinic, Silkeborg Regional Hospital, structured with inspiration from the Template for Intervention Description and Replication (TIDieR) checklist and guide [36].


**1.** Brief name	ReGa – Integrated Rheumatology-Gastroenterology Clinic

**2.** Why	The rationale for the ReGa clinic is based on the organisational need to create continuity in patient pathways for patients with multiple autoimmune diseases, the clinical need to coordinate treatment for the patient group and the patient’s needs for an individualised person-centred approach.

	The Danish healthcare system is highly specialised. Systematic challenges arise in particular for patients with multiple chronic diseases, where patients typically are assigned to several distinct departments, with each disease treated separately by different specialists [2]. The presence of one autoimmune disease increases the risk of developing multiple autoimmune diseases, such as in patients with both IBD and IJD. This combination often requires complex treatment with contraindicated medications as a medical challenge [16]. The co-occurrence also affects several aspects of patients’ lives, including challenges in family life, work life and treatment in healthcare [8].

	To address these challenges, several different elements were considered. A multidisciplinary approach with both specialties was chosen to ensure coherence and the right treatment. Furthermore, the clinic was inspired by the person-centred care model, where a human and value-based approach involves the patient as an active part of their care/treatment and in the decision-making process [37].

	The focus is on the patient’s problems and not their diagnoses.

	ReGa is thus an attempt to adapt the system to the patients. By doing so, patients’ needs for coordination of care and treatment are accommodated while focusing on the individual rather than the disease. Another assumption is a temporal benefit for both patients and professionals, as care for both conditions is provided simultaneously.

**3.** What – materials	A consultation room in the Department of Rheumatology.

**4.** What – procedures	Patients and any accompanying relatives are met in the waiting area by the clinic’s rheumatologist. The rheumatologist retrieves the patient, allowing for an immediate observation and impression of physical functioning, in particular, how the patient rises from the chair and walks into the consultation room.

	The gastroenterologist starts by asking questions about the patient’s inflammatory bowel disease. Subsequently, the rheumatologist asks questions about the patient’s inflammatory rheumatic disease and performs joint examinations if necessary.

	The consultation flow typically reflect the chronological order in which the patient’s conditions were diagnosed, as most have lived with IBD for many years prior to developing a rheumatic disease.

	The doctors occasionally supplement one another, and the consultation unfolds as a shared dialogue all participants. The conversation with the patient typically includes disease status, results from tests or scans, and follow-up or adjustment of medical treatment. Using illustrations of the intestinal and skeletal systems, the doctors provide patient education on their conditions, including how and why their autoimmune diseases develop. The conversations also include discussion of the patient’s work and family life.

**5.** Who provided	The resources in ReGa consist of an experienced doctor and a nurse from both gastroenterology and rheumatology departments. Booking and coordinating appointments is managed by a nurse from the rheumatology department.

**6.** How	The multidisciplinary approach consists of a face-to-face model, where the two doctors see the patients at the same time, in the same room. Occasionally, contact is also made by telephone, either on the patient’s or healthcare professional’s initiative.

**7.** Where	The ReGa Clinic takes place at the outpatient Department of Rheumatology at Medical Diagnostic Centre, Silkeborg Regional Hospital, Denmark.

	Patients are primarily referred from the rheumatology and gastroenterology departments at Silkeborg Regional Hospital. Referrals are occasionally also sent from other hospitals within and outside the region, and in some cases, patients request enrolment after learning about the clinic.

**8.** When and How much	Newly referred patients are scheduled for one hour, while follow-up visits last half an hour. This allows additional time to conduct thorough examinations within each speciality and develop a joint treatment plan. Based on the first assessment, patients are referred to the gastroenterology or rheumatology department or invited to continue in the ReGa clinic.

	The clinic is open for patients on a monthly basis.

	Patients can reach the specialists by contacting the rheumatology or gastroenterology departments from 8 AM to 4 PM on weekdays.

**9.** Tailoring	The number of clinic visits and the duration of treatment depend on the individual patient and the complexity of their diseases. The course of treatment is therefore, based on medical need and specialist assessment.

**10.** Modifications	During the pilot phase, an increasing need became apparent as referral rates increased. Consequently, the number of appointments was expanded along with the addition of two nurses, and the ReGa clinic now sees patients regularly every month.


The ReGa clinic was established through the initiative of specialists in the departments of rheumatology and gastroenterology. The clinic focuses on patients with multiple autoimmune diseases and the complex treatment they often require. It provides diagnosis and multidisciplinary treatment for adult patients diagnosed (or suspected) with both inflammatory bowel and joint diseases. The ReGa clinic adopts a multidisciplinary approach, facilitating integrated, face-to-face care for patients [35]. In this model, a rheumatologist and a gastroenterologist collaborate by seeing patients together, providing a cohesive clinical experience. The resources in ReGa are primarily a doctor and a nurse from each speciality who consult patients monthly. The number and duration of consultations depend on the individual patient and the complexity of their diseases and course of treatment. Most referrals to the ReGa Clinic come from internal departments at Silkeborg Regional Hospital, particularly the rheumatology and gastroenterology departments. Occasional referrals also come from other regional hospitals based on patients’ requests following recommendations about the clinic.

## Data collection

The study included all patients seen at the ReGa clinic between 2018 and 2022 and explored the experiences of a subset of these patients using a qualitative research approach. Quantitative data were used to provide a descriptive overview of the full patient population, while qualitative interviews offered in-depth insight into patients’ experiences with the integrated care model.

### Description of the patients

Data on patient characteristics including gender, age, diagnosis, number of outpatient visits, missing scheduled visits, hospital admissions, and length of care pathways, were extracted from the electronic patient record in the Central Denmark Region between January 2018 and December 2022.

Descriptive statistics were used to summarise the characteristics of the population and their contacts with the hospital indicated by numbers (n) and proportions (%), or mean and min-max ranges.

Data was analysed using Stata, version 17 (StataCorp, College Station, TX).

### Patient experiences

Qualitative data was generated through six months of participant observation and individual semi-structured interviews from November 2022 to March 2023. This process included observing clinic operations and accompanying patients, guiding our understanding of ReGa as an integrated care case, and preparing the interview guide. Interview participants were recruited through consultations on two clinic days. Of the 10 patients who agreed to participate, nine were interviewed, and one failed to return to participate, presumably due to geographical reasons. To ensure convenience and accommodate preferences, patients were offered a choice of interview settings: at home, at the hospital, or virtually. This resulted in three interviews conducted at patients’ homes, three at the hospital, two via Zoom, and one by telephone. While the interview setting may influence dynamics or responses, no significant differences in interview quality or analytical usefulness were observed across contexts.

The interviews were audio-recorded and lasted 25–74 minutes. Data were transcribed verbatim and analysed using Framework Analysis [38]. With a step-by-step approach, interpretation and conclusions were reviewed at each completed step, providing a structure to analyse data by participants (rows) and themes (columns), enabling comparison and contrast of data both within and across participants and themes. Coding was conducted in a Word document encompassing transcriptions from all nine interviews. Relevant sections within the text were identified and organised under corresponding themes. Three to seven subthemes were subsequently identified for each main theme. Regular meetings in the research group facilitated a critical exploration of participant responses. Themes were primarily discussed and agreed upon between SHJJ and MF.

Finally, Haggerty et al.’s definition of continuity of care provided the conceptual framework for examining patient experiences in the ReGa clinic [39]. The definition was particularly useful as patient perspectives and experiences constitute key elements in understanding whether an intervention improves continuity of care and which factors may be significant. The definition identifies three types of continuity in patient care: *informational, management*, and *relational*. If all three dimensions are met, patients typically experience predictability, safety, and continuity of care. Notably, this definition of continuity considers patients’ needs and life circumstances. It also emphasises that for continuity to occur, care must be experienced as such by patients, highlighting that interventions designed to improve continuity of care are not inherently sufficient [39]. Data were analysed with these three dimensions in mind but performed as an iterative process encompassing empirical material, thematic analysis, and the conceptual framework.

## Ethical considerations

Following the Danish General Data Protection Regulation (GDPR), permission was granted by the hospital management to extract data from medical records. Data were transferred from the electronic medical record to MidtX (a secure and authorised regional digital platform). Informed consent was obtained from patients before participating in the interviews.

To mitigate the risk that participants might respond more favourably due to a perceived link to future care, they were informed that their involvement was entirely voluntary and that their responses would have no consequences for their current or future treatment. Furthermore, interviews were conducted by a researcher independent of the clinical care team to reduce potential influence.

## Results

### Patient characteristics

**Figure 1:**
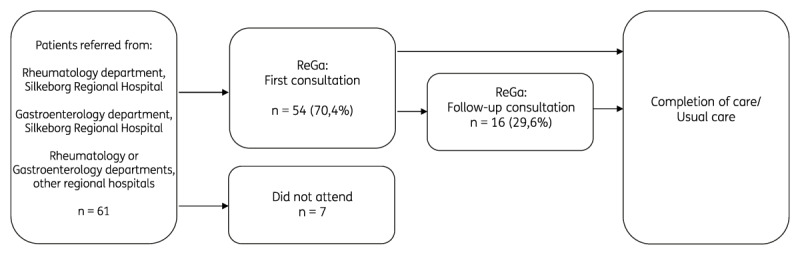
Flowchart of Patient Pathways in the Rheumatology-Gastroenterology clinic, Silkeborg Regional Hospital.

Of the 61 patients referred to the ReGa Clinic between January 2018 and December 2022, 11,48% did not attend their appointments, leaving 54 patients to be included in further analysis (Figure 1). A large majority of patients (70,4%) were seen once, and 16 patients (29,6%) were seen more than once with a maximum of 6 visits (Figure 1).

The majority of patients were male (57,4%), and the largest proportion were aged 40–49 (27,8%) (Table 2). The 54 patients attending the ReGa clinic had an average of 29,6 outpatient contacts with the rheumatology clinic and/or gastroenterology clinic, ranging from one to 67. The number of outpatient contacts originated from patients’ primary department, as participants included in the study were affiliated with either the rheumatological or gastroenterological department or both while concurrently receiving care at the ReGa clinic. For most patients (83,3%) care was completed within the first year. Fifteen patients (27,8%) were admitted to the hospital during the study period, with a total of 38 admissions. These admissions were related to either gastroenterological or rheumatological conditions, including inflammatory joint and bowel diseases. The majority of admissions were related to gastroenterology (84%), with the remaining related to rheumatology (16%).

**Table 2:** Characteristics of patients attending The Rheumatology-Gastroenterology clinic, Silkeborg Regional Hospital n = 54.


**Gender (male), n (%)**	31 (57.4)

**Age at baseline (years), n (%)**	

20–29	8 (14.8)

30–39	12 (22.2)

40–49	15 (27.8)

50–59	12 (22.2)

60+	7 (13)

**Number of outpatient visits in ReGa pr. patient, n (%)**	

1	38 (70.4)

2–4	14 (25.9)

5–6	2 (3.7)

**Length of patient pathway in years, n (%)**	

<1	45 (83.3)

1	2 (3.7)

2	5 (9.3)

3	2 (3.7)

**Total outpatient contacts during the study period, n**	1601

Number of outpatient contacts pr. patient, mean (min-max)	29.6 (1–67)

Contacts related to gastroenterology	905 (56.5)

Contracts related to rheumatology	696 (43.5)

**Proportion of patients admitted during study period, n (%)**	15 (27.8)

**Admissions in total, n**	38

Admissions related to gastroenterology, n (%)	32 (84)

Admissions related to rheumatology, n (%)	6 (16)


The interviewed group consisted of five men and four women. Ages ranged from 30 to 86 years. Employment status was evenly distributed across full-time employment, part-time employment, and public income support.

### Patient experiences

Guided by Haggerty et al.’s definition of continuity in care, our analysis identified three main themes relevant to patient experiences of the ReGa clinic. These themes were elucidated through the three dimensions of continuity — informational, management, and relational — serving as lenses to explore and convey these experiences.

#### Informational continuity

Informational continuity was present through patients’ descriptions of how the multidisciplinary approach provided information between all parties involved, ensuring that health professionals had sufficient knowledge of information about treatment, medication, and personal preferences.

The multidisciplinary approach with multiple specialists was experienced as holistic and integrated care by most patients. This approach allowed for simultaneous treatment of both diseases and the opportunity for patients to ask questions related to both rheumatological and gastroenterological conditions. Having this option, patients left consultations with a sense of being in control and “having it all together,” thereby expressing strong informational continuity (Table 3). For most patients, this meant that information was not lost across departments, as previously experienced. One female patient described how the presence of both doctors prevented miscommunication and general antagonism across departments (Table 3).

**Table 3:** Interview quotes from patients, Rheumatology-Gastroenterology clinic, Silkeborg Regional Hospital.


MAIN THEMES	PATIENT QUOTES

**Informational continuity**	If you visit different clinics several times, questions can easily be forgotten (..); the thing is that here [in the ReGa Clinic], nothing is lost, nothing is wasted, and as a patient, you get a lot out of it by having them both. – Male 51 years old, Crohn’s disease and Rheumatoid arthritis. It’s so much easier when they sit and talk about things together. Instead of me having to explain what happened in one place when I got to the other place (…) There are often things that don’t quite come together properly when you’re navigating across systems. – Female 30 years old, Crohn’s disease and Rheumatoid arthritis. The most important thing is being able to make contact – that there’s a connection, that you can reach a doctor or a nurse, and that’s something I have always experienced (…). For me, the most important thing is that when something has come up, there has been someone at the other end who has helped. – Female 46 years old, Crohn’s disease and Rheumatoid arthritis.

**Management continuity**	I get that percentage fewer appointments because some of it has been merged (…) I think it works overall, this merging thing, but when you visit almost all departments except fertility or the women’s ward, there could be more of it. – Male 37 years old, Crohn’s disease, Rheumatoid arthritis, and another chronic disease. When you have a dual diagnosis, it’s important to get help understanding the connections and challenges involved, and a joint consultation really helps with that. Also, it’s a great help to be able to manage with fewer visits. – Male 86 years old, Ulcerative Colitis and Spondyloarthritis. I actually have an hour’s drive to get here [the hospital], so it’s also a matter of time. You have to go to the hospital so often when you have these conditions, especially when they keep haunting you. So, for me, it’s really wonderful that they can sit together and discuss it. – Female 30 years old, Crohn’s disease and Rheumatoid arthritis.

**Relational continuity**	You can have a conversation that is completely down to earth while not being unserious at all; on the contrary, it is a feeling that you are taken seriously while, at the same time, you do not feel that you have entered the rulers of some kingdom you did not know existed, so in that way, I feel very much on the same page with them. – Male 56 years old, Ulcerative colitis and Rheumatoid arthritis. With chronic disorders, you need to feel secure in relation to the treatment you receive, and you achieve this security, by being confident about their decision that you should have it [medication], that it is right, and now that it is two specialists, who have agreed that this is something for me, then of course I experience a special security. – Male 86 years old, Ulcerative Colitis and Spondyloarthritis. You kind of feel like you get a… not a relationship, but, you know, there’s this sense of knowing each other and talking, and that makes you feel a bit more comfortable with it (…) For me, it’s made a huge difference that it’s the same [healthcare professionals], because we kind of know each other, and they know me and who I am, and I think that means a lot. – Female 32 years old, Ulcerative colitis and Rheumatoid arthritis.


Some patients preferred an expanded team involving more healthcare professionals, such as an endocrinologist, to ensure adequate information and address additional comorbidities and chronic diseases. Several newly referred patients expressed uncertainty about when or how often they were expected to attend the ReGa clinic. They lacked information about when they would be called in for new consultations, medical treatment, or receive various test results, indicating a need for more initial information about the structure of the clinic.

#### Management continuity

Concerning management continuity, patients provided insight into the significance of combining the rheumatology and gastroenterology clinics and identified potential organisational barriers.

Patients unanimously agreed on the need for integrated treatment for their interrelated diseases, a goal the clinic adeptly fulfilled by combining specialities. The integration was profoundly beneficial, enhancing medical care—a crucial factor for patients, considering the chronicity of their conditions and the complexities previously experienced in achieving well-managed treatment and health.

Patients expressed their satisfaction to such an extent that they wished the clinic were expanded. The desire for expansion was described by a male patient who, in addition to inflammatory joint and bowel disease, also suffered from another chronic disease. According to the patient, it would help to bring his appointments even closer together, as he was also treated by other health professionals (Table 3). The ReGa clinic may thus represent a small piece of a larger puzzle for patients who also attended other hospital clinics. While it was perceived as progress and a significant step in addressing the broader issue of fragmented patient pathways, management continuity was, for some patients, still lacking.

The combination of specialities resulted in an experience of convenience for patients. The perceived convenience was attributed to temporal and logistical factors, as patients expressed high satisfaction with the reduced number of hospital visits and the uncompromised quality of care. Patients emphasised the burden of frequent hospital visits typically associated with multiple chronic conditions, and greatly appreciated having appointments combined. Numerous patients used the expression “killing two birds with one stone” to describe the organisation of the ReGa clinic, adeptly capturing their attitude of convenience and optimism regarding the integrated practice.

Several patients highlighted various organisational barriers in the ReGa clinic’s management continuity and suggested ways to accommodate these. Firstly, patients requested that blood and stool samples were analysed simultaneously, like combined consultations, to avoid redundancy. At the time of the study, patients had to deliver samples at separate clinics prior to each consultation, which was experienced as both inconvenient and time-consuming, as it also appeared to constitute a large part of the treatment and patient pathway. Secondly, some patients found it challenging to contact the clinic outside of scheduled appointments, as contact was limited to weekday office hours through either the rheumatology or gastroenterology department.

Patient-reported outcomes (online questionnaires) were used before appointments, providing specialists with information on patients’ symptoms and well-being. Most, but not all, patients understood and managed using the questionnaires. However, it was experienced as challenging that separate questionnaires were required for each condition, i.e., patients had to report in two questionnaires, one for their rheumatic disease and one for their bowel disease. A single database for reporting symptoms was repeatedly mentioned to improve management continuity in the ReGa clinic.

#### Relational continuity

As characterised by relational continuity, patients highlighted the importance of feeling secure and involved in their treatment, which the specialists in the ReGa clinic and their interprofessional relationship enabled.

Being treated by the same specialists at the same time when visiting the clinic greatly contributed to patients’ sense of security. Patients highlighted that the specialists knew them well, avoiding the need to introduce themselves and their disease history repeatedly. At the same time, this led to a coordinated and agreed treatment plan among the treating specialists, which patients experienced as comforting and reassuring. With both diseases considered at the same time, patients avoided concerns about whether treatment for one disease could potentially have an adverse effect on the other disease (Table 3).

All patients emphasised their positive experience with the presence of two specialists. They appreciated listening to the specialists discussing their treatment plan, which contributed to a sense of involvement in their treatment and related decision-making, in contrast to previous experiences. The consultation form also met patients’ need to be assessed and examined from multiple perspectives, which one patient found particularly meaningful. This patient sought a referral to the clinic following a taxing journey through the healthcare system. His new-onset arthritis symptoms had previously been dismissed as being related to his bowel disease, a possible link he felt was important to investigate and take seriously (Table 3).

Several patients described how the patient-centred care made them feel valued and an equal part of the treatment. The feelings were expressed through patients’ experiences of consideration for things other than medical and disease-specific issues. Patients described how the specialists in the ReGa clinic had been immensely helpful in regards to their work and private lives, adapting the treatment to personal preferences and needs, thus acknowledging that life with multiple chronic diseases extends beyond illness.

Finally, relational continuity was experienced in the strong internal collaboration between health professionals, leading to a comfortable atmosphere. This supportive setting was particularly important to one female patient who generally found it difficult to attend hospital appointments due to anxiety about medical consultations. Another, who had a very positive experience of collaboration between the health professionals, saw it as a professional surplus that greatly benefited him and other patients. Thus, relational continuity was considered particularly important for all patients, and strong collaboration among health professionals was experienced more significant than organisational or management factors. Several participants also described how it may benefit the health professionals themselves, as the ReGa clinic provided an opportunity to learn about the other speciality and exchange knowledge and experiences. This serves as a good example of how the conventional compartmentalisation of expertise across medical specialities and hospital departments can be addressed, thereby promoting interdisciplinary learning.

## Discussion

The ReGa clinic involves coordination of treatment, knowledge sharing and continuity of care for patients with multiple autoimmune diseases. Patients at the clinic are characterised by being predominantly of working age, and although most have few contacts with the ReGa clinic, they generally have many outpatient contacts with the hospital, most of which are related to IBD. Patients viewed the relational aspect as the cornerstone of continuity. Still, it was clear that information and management continuity were also critical components of the overall experience of continuity of care. With all three components present, ReGa is perceived as an intervention that contributes to an overall experience of continuity in care for patients with multiple autoimmune diseases.

Research on integrated care and combined clinics has predominantly centred on disease-specific outcomes with quantitative measures and integration of dermatology and rheumatology for patients with psoriasis and psoriatic arthritis [20,21,22,23,24,25,26,27,28,29,30]. By exploring gastroenterology in combination with rheumatology and incorporating patient experience using qualitative methods, this study extends the focus of the existing literature [31,32,33,34]. Despite diverse approaches and methodologies, earlier studies on combining medical specialities shared our understanding that the combination and close collaboration among specialists improve treatment, make a difference for patients, and result in the delivery of enhanced integrated care [20,21,22,23,24,25,26,28,31,32,33,35]. Our approach highlighted the key components of the intervention and the processes involved in delivering it. As such, the in-depth exploration of clinic operations and patient interactions may provide valuable insights for replicating and implementing similar models in new contexts. Our findings complement the prevailing diagnostic focus within the field of autoimmunity and patient pathways, which advocates collaboration between specialties to ensure the best possible care for patients with multiple autoimmune diseases [32,35,40].

This study contributes to a greater focus on the patient perspective in multidisciplinary healthcare research, revealing patients’ needs, experiences and expectations [35]. The experiences provided insights into what works for patients with autoimmune diseases. Patients largely experienced responsibility, cooperation, and recognition in the ReGa clinic, contrasting previous studies that found collaboration and coordination between specialists problematic [7,8]. The preponderance of positive descriptions of patient-centred care and a multidisciplinary approach emphasises that collaborative practice at the ReGa clinic is experienced as both rare and a contribution to improved continuity of care.

We found that relational continuity was particularly important for the experience of coherence, confirming the claim that intimate micro-level situations are essential for continuity of care [41,42,43,44]. This finding also consistent with literature highlighting the importance of interprofessional practice and relational coordination, arguing that this ultimately leads to improved quality of care [45,46].

The patient’s high level of satisfaction with the ReGa Clinic was also reflected in their encouragement of expanding the intervention. Being a patient at the ReGa Clinic eased managing multiple chronic conditions, as aligned visits resulted in a sense of security, involvement, and treatment optimisation. The collaboration of specialties spared the patient from the burden of coordinating and forwarding information between clinics. It was reassuring to meet the same healthcare professionals from time to time. Altogether, these factors contribute to an improved and more continuous patient pathway, and the holistic and inclusive character of the approach in ReGa was valued by its patients.

Our findings highlight that compartmentalisation still exists within the clinic’s communication and organisational structures, revealing potential areas for improvement of the intervention. The results indicate that future efforts should improve information levels and align expectations to avoid initial confusion and uncertainty for newly referred patients. Patients with more chronic conditions than those treated at the ReGa clinic appreciated the intervention but requested further integration and involvement of additional specialties. In addition, it would be beneficial for the experience of continuity if organisational processes more clearly reflected the clinic’s integrated focus, e.g., by consolidating blood and stool samples in one location and merging online patient-reported questionnaires into a unified system. This would acknowledge that from the patient’s perspective, care pathways extend beyond the consultation itself, encompassing a broader journey that includes numerous activities, events and contacts occurring both before and after the clinic visit [47]. This may also reflect a tendency wherein patients generally place high expectations on the healthcare system [48,49]. At the same time, navigating complex organisational structures that are not always transparent from a patient perspective may potentially result in experiences perceived as less satisfactory [50]. This emphasises how patients view the healthcare system differently from healthcare professionals.

The patients’ positive approach to consolidating and reducing hospital contacts aligned well with the fact that a significant number of the patients were of working age, where numerous appointments had previously impacted their daily lives negatively. The observation that care was completed for the majority of patients within the first year, and most patients have only a single visit, could be indicative of the clinic’s effectiveness in achieving stabilisation and clarification, thereby enabling a transition to regular outpatient care for these patients, while more complex cases are retained at the clinic for a longer period. Despite the brevity of these visits, the consultations were experienced as crucial and invaluable for patients, providing a sense of calm, confidence, and reassurance regarding their conditions and further treatment.

## Strengths and limitations

The study used both quantitative and qualitative data, incorporating patient characteristics and experiences, to provide a nuanced understanding of the study population and intervention. This approach and the use of the TIDieR checklist enabled a comprehensive description of the ReGa clinic and the patients attending it, facilitating transferability to other contexts. Furthermore, we employed Haggerty et al.’s definition as a conceptual framework to interpret and understand continuity of care [39] in the ReGa clinic. The framework guided both our analysis and interpretation of data, allowing integration of our findings with existing literature in the field.

The active participation of patients in examining the intervention stands as a strength of the study. However, it should be acknowledged that the interviewed patients only represented those who visited the clinic multiple times. This group may differ in their experiences or levels of satisfaction compared to patients who attended only once. Frequent visitors might be more familiar with the clinic’s procedures or have developed stronger relationships with the healthcare professionals. As a descriptive study, it lacks findings on effectiveness and costs. Despite the study’s primary objective not being to assess the impact of the clinic on reduced visits, the qualitative findings indicate an enhancement in effectiveness through collaborative consultations. Nevertheless, in the absence of quantitative outcome measures, it is not possible to draw definitive conclusions about the effectiveness of the intervention. To strengthen the evidence, future research should evaluate the clinic’s impact on clinical effectiveness, costs and resource allocation.

Furthermore, the ReGa clinic was not established as a research intervention. Instead, the intervention arose from an identified need through clinical experience and prior literature, with a strong interest in developing an integrated solution. We present a case description and believe that elements of the ReGa clinic can be an inspiration to future care organisations for patients with multiple autoimmune diseases, and more broadly, patients with multimorbidity. Finally, many patients with autoimmune diseases are undiagnosed, indicating that the ReGa clinic could increase its impact and achieve even greater potential by focusing on screening for, e.g., rheumatic diseases in IBD patients.

## Conclusions

Combining specialities led to an experience of improved continuity of care for patients with multiple autoimmune diseases. The integrated practice enhanced the management of patients’ complex treatment, and the multidisciplinary approach ensured information and collaboration between healthcare professionals. In particular, the findings emphasised that relational continuity, including interprofessional collaboration, was essential in promoting an experience of continuity of care. The significant number of working-age patients also emphasised the importance of combined appointments leading to fewer hospital visits, which may enhance opportunities to maintain employment and improve quality of life. The results highlight the potential to bridge healthcare gaps for patient groups affected by fragmented care across departments and increasing specialisation in healthcare, with possible benefits for other patient groups with comorbid conditions.

More research is needed on the advantages of combined clinics and other aspects of collaborative healthcare practices, including healthcare professionals’ experiences and health economic analysis. Future research could build on the insights gained from the ReGa clinic, as it offers a solution to the prevailing challenge of lacking coordination and continuity in care for patients with multiple autoimmune and chronic diseases.

## Figures and Tables

**Table:** 

IBD:	Inflammatory Bowel Disease
IJD:	Inflammatory Joint Disease
ReGa:	Rheumatology-Gastroenterology Clinic
